# Counselling, Case Management and Health Promotion for People Living with HIV/AIDS: An Overview of Systematic Reviews

**DOI:** 10.1007/s10461-012-0283-1

**Published:** 2012-09-09

**Authors:** Michael G. Wilson, Winston Husbands, Lydia Makoroka, Sergio Rueda, Nicole R. Greenspan, Angela Eady, Le-Ann Dolan, Rick Kennedy, Jessica Cattaneo, Sean Rourke

**Affiliations:** 1 McMaster Health Forum, McMaster University, Hamilton, Canada; 2 Centre for Health Economics and Policy Analysis, McMaster University, Hamilton, Canada; 3 Department of Clinical Epidemiology and Biostatistics, McMaster University, Hamilton, Canada; 4 Ontario HIV Treatment Network, Toronto, Canada; 5 AIDS Committee of Toronto, Toronto, Canada; 6 University of Toronto, Toronto, Canada; 7 Health Research Methodology Programme, McMaster University, Hamilton, Canada; 8 Canadian Working Group on HIV and Rehabilitation, Toronto, Canada; 9 Ontario AIDS Network, Toronto, Canada; 10 Centre for Research on Inner City Health, St. Michael’s Hospital, Toronto, Canada

**Keywords:** HIV, Systematic review, Case management, Health promotion, Counselling

## Abstract

Our objective was to identify all existing systematic reviews related to counselling, case management and health promotion for people living with HIV/AIDS. For the reviews identified, we assessed the quality and local applicability to support evidence-informed policy and practice. We searched 12 electronic databases and two reviewers independently assessed the 5,398 references retrieved from our searches and included 18 systematic reviews. Each review was categorized according to the topic(s) addressed, quality appraised and summarized by extracting key messages, the year searches were last completed and the countries in which included studies were conducted. Twelve reviews address topics related to counselling and case management (mean quality score of 6.5/11). Eight reviews (mean quality score of 6/11) address topics related to health promotion (two address both domains). The findings from this overview of systematic reviews provide a useful resource for supporting the development and delivery of evidence-informed support services in community settings.

**Electronic supplementary material:**

The online version of this article (doi:10.1007/s10461-012-0283-1) contains supplementary material, which is available to authorized users.

## Resumen

Nuestro objetivo fue identificar todas las revisiones sistemáticas relacionadas al asesoramiento, el manejo de casos y la promoción de la salud en personas que viven con el VIH/SIDA. En las revisiones identificadas, evaluamos la calidad y aplicabilidad local para respaldar políticas y practicas informadas por la evidencia. Realizamos búsquedas en 12 bases de datos electrónicas, dos evaluadores revisaron de forma independiente las 5398 referencias identificadas e incluimos 18 revisiones sistemáticas. Cada una de las revisiones incluidas fue clasificada de acuerdo al tema presentado, valorada en terminos de su calidad, y resumida en base a la extracción de los mensajes principales, el ultimo año en que la busqueda tuvo lugar y los países incluidos en los estudios que formaron parte de la revisión. Doce revisiones abordan temas relacionados con el asesoramiento y el manejo de los casos (con un promedio de puntuación de calidad de 6.5/11). Ocho revisiones (con un promedio de puntuación de calidad de 6/11) abordan temas relacionados con la promoción de la salud (dos revisiones abordan ambos dominios). Los resultados de este compendio de revisiones sistemáticas constituyen un recurso útil para respaldar el desarrollo y la prestación de servicios de apoyo debidamente informados por la evidencia en contextos comunitarios.

## Introduction

The cornerstones of community support services for people living with HIV/AIDS (PHAs) are case management, counselling and health promotion. Case management focuses on helping service users to identify their unmet needs, and linking them with the required health and social services to achieve desired outcomes [1–3]. After an initial assessment of needs, the case manager and service user collaborate on a system of referrals, monitoring, follow-up assessment and advocacy to ensure positive outcomes. Needs may vary in scope from those that are considered basic (e.g., food and shelter) to those that may be more remote but instrumental to achieving basic needs (e.g., legal services) [4]. Psycho-social counselling may be an important component of case management but is also a stand-alone intervention. Gerbert et al. [5] have noted that counselling is one of the most powerful ways to address the psycho-social aspects of HIV, which include managing risky behaviours, coping and social support, depression and treatment adherence [5]. Counselling and case management typically focus on individuals, but health promotion may have a distinctly community focus.

Health promotion is “the combination of educational and environmental supports for actions and conditions of living conducive to health” [6]; and is a “process of enabling people to take control over, and to improve, their health” [7]. This includes promoting behaviour change to achieve better health, as well as helping people and communities negotiate or dismantle the barriers to good health. Health promotion therefore includes an explicit concern with structural factors that impact health and access to health, which places community engagement and community development as intrinsic components of its mission [8].

Community-based organizations are integral to delivering these types of support services and programs to help address the increasingly complex health-related and social issues experienced by PHAs [9, 10]. These support services can impact the health of PHAs and those at risk for HIV by helping to prevent future HIV infections and addressing powerful social determinants of health such as increased social support and integration. In addition, offering HIV/AIDS support services through community-based organizations helps ensure that services are attuned to the specific needs of the communities they serve. However, most efforts towards supporting the use of research evidence have focused on clinical and prevention services, with far less effort devoted to mobilizing knowledge about effective practices in community-based organizations that provide essential on-the-ground support for PHAs.

Even though there is some debate about what constitutes “evidence” and the best approaches to effectively translate or transfer evidence to practitioners [11–13], there is general agreement that health practitioners need access to the best available research evidence to inform and support their practice [14–20]. In general, evidence-based practice (or evidence-informed decision-making) refers to practitioners’ use of standards of care whose effectiveness has been demonstrated through research evidence. In other words, decisions about care and treatment should be informed by the best available research evidence. Service providers working within health systems may improve patient, client and service user outcomes. This may then result in more efficient use of health system resources by applying care and treatment options that have been shown to be effective at improving health outcomes.

Systematic reviews are a key source of research evidence for supporting evidence-informed practice at the community level for several reasons. First, using systematic reviews is an efficient use of time for busy managers and service providers because all information on a specific topic has already been identified, selected, appraised, and synthesized in one document [21]. Research users are also less likely to be misled by findings from systematic reviews as compared to other forms of research (e.g., a single experimental study). Also, due to the gains in precision associated with synthesizing multiple studies, systematic reviews may inspire greater confidence in research findings among those who use research to support program and policy development [21]. Lastly, systematic reviews are increasingly incorporating a broader spectrum of research evidence (e.g., syntheses of qualitative evidence) [22–29] to answer questions beyond the standard effectiveness of interventions. This broader range of applications (e.g., issues related to the cost-effectiveness of interventions, and how and why interventions work) increases the relevance of systematic reviews to a wider target audience [21, 30].

To support the delivery of evidence-informed support services in community settings, we conducted an overview of systematic reviews. Our general aim was to mobilize relevant and high-quality research evidence related to counselling, case management and health promotion for PHAs. Our specific objectives were to: (1) identify and assess the quality and local applicability of systematic reviews in each of the two fundamental domains of support services (i.e., counselling and case management, and health promotion); (2) develop user-friendly summaries of the key findings and recommendations from each included systematic review: and (3) broadly disseminate the summaries to community-based organizations that service PHAs.

## Methods

We searched 12 electronic databases in April 2009 using a search strategy designed to optimize the retrieval of systematic reviews (the search strategy is provided in Appendix A, available as a supplement to the online version of this article). We supplemented this by scanning the reference lists of included systematic reviews. Two teams of reviewers (LM and a research assistant, and LM and WH) independently assessed the titles and abstracts for inclusion. Disagreements were resolved by consensus and a third reviewer (MGW) made a final decision where no consensus could be reached. At this initial stage of reviewing, references were included if they were either a systematic reviews or a primary research study and addressed a topic related to counselling, case management and/or health promotion for people living with HIV/AIDS. Two teams of independent reviewers (LM & WH, and LM & MGW) then assessed the references included after the initial scoping stage to identify the systematic reviews meeting our inclusion criteria.

We retrieved the full-text of all included systematic reviews and two reviewers (WH and LM) conducted a final inclusion assessment. Next, two of us (MGW and SR) conducted independent appraisals of the methodological quality of each included systematic review using the AMSTAR (A MeaSurement Tool to Assess Reviews) instrument [31]. AMSTAR demonstrates both strong face and content validity [31], and is regarded as an optimal approach for assessing the quality of systematic reviews [32, 33]. This scale produces a quality score between 0 and 11, representing low (scores between 0 and 3), medium (scores between 4 and 7) and high (scores between 8 and 11) quality systematic reviews. We did not assess the quality of the studies included each review, which is typically conducted as part of the reviews themselves. We therefore deemed it more appropriate to provide a gauge to the quality of the methods used by each systematic review to synthesize the primary studies included in them. Using the scores of methodological quality from each review, we calculated average quality scores for each topic domain. We standardized the mean quality score by converting each score into a percentage, which we used to calculate the mean score out of 11. This standardization was necessary due to the fact that the denominators for quality appraisal scores can vary using the AMSTAR tool when a question is deemed to be ‘Not applicable’.

One of us (MGW) then categorized reviews by topic and extracted key messages, the year searches were last completed and the countries in which included studies were conducted (categorized by high and low- and middle-income countries). This work was then checked by three members of the team (WH, SR and LM) for accuracy.

Lastly, we developed user-friendly summaries for each included systematic review. We used an approach developed through a recent study with 31 executive directors and program managers of Canadian community-based organizations from the HIV/AIDS, mental health and addictions and diabetes sectors [34]. The user-friendly summaries are presented in a format that provides: (1) an outline of the topic of the review, a plain language summary and a bulleted list of key messages summary; (2) an outline of the benefits, harms and costs of the intervention, program or service evaluated in the review; and (3) relevant equity and local applicability considerations. All of the user-friendly summaries are available through an HIV/AIDS evidence service (SHARE—Synthesized HIV/AIDS Research Evidence) (http://www.hivevidence.org/SHARE/ResourcesSummaries.aspx) [35].

## Results

**Fig. 1:**
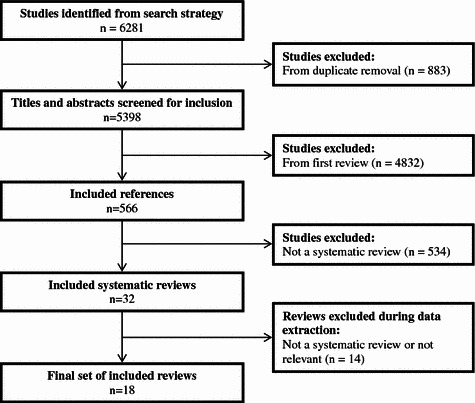
Flow diagram of study selection

Our searches yielded 5,398 references from which we excluded 4,832 based on title and abstract review and 545 after assessing the full-text articles, leaving 18 systematic reviews (12 conducted meta-analyses) that met our inclusion criteria. The study selection process is outlined in Fig. 1.

Twelve of these reviews address topics related to counselling and case management, which have a mean quality score of 6.7/11 (see Table 1). Eight reviews address topics related to health promotion (see Tables 1, 2) which have a mean quality score of 5.9/11. Three address both domains but are presented only in Table 1 (each is identified under footnote a) but are included in the average quality calculations for both domains. Most of the systematic reviews (11 of 18) were published since 2005, all included studies from high-income countries and five include studies from low- and middle-income countries.

**Table 1:** Included systematic reviews about counselling and case management

Review	Domain/topics studied	Focus of review	Key findings	Year of last search	AMSTAR (quality) rating	Countries in which included studies were conducted
Handford et al. [36]	Organization (including case management) and setting of care	To evaluate the association between the setting and organization of care and outcomes for people living with HIV/AIDS	Centralizing care in high concentration/high volume centres could lead to improved outcomes including mortality, but this evidence is mixed and limited to developed country settings Case management may be associated with improved outcomes but the limited number of studies and the varying definitions of case management leave considerable doubt about how best to implement such programs Multidisciplinary and multi-faceted treatments, health information systems and extended hours of operation are promising interventions but evidence about their effectiveness is so far lacking	2002	10/10	Not reported
Himelhoch et al. [39]^b^	Counselling Psychotherapy Depression	To examine the efficacy of group psychotherapy treatment among HIV infected with depressive symptoms	Group therapy (particularly group cognitive behavioral therapy) may be efficacious in treating depressive symptoms among PHAs; however, the underrepresentation of women in the included studies limits the generalizability of the reviews findings Because women may be at risk for depression and are an emerging population at risk for HIV (in high income countries), future studies should address this issue	2006	9/11	Low- and middle-income countries (0) High-income countries (8): United States (6); Netherlands (1); China (Hong Kong) (1)
Rueda et al. [42]^b^	Counselling Medication management and adherence Sexual health/risk behaviours	To assess the effectiveness of patient support and education to improve adherence to highly active antiretroviral therapy	Intervention features that were linked to successful adherence outcomes included those: targeting practical medication management skills, administered to individuals vs. groups, and delivered over 12 weeks or more; but not those targeting more complex psychological constructs or targeting marginalized populations such as women, Latinos, or patients with a past history of alcoholism This review did not find any studies that examined the effectiveness of provider-level interventions (e.g., those interventions that provide feedback to practitioners) and system-level interventions (e.g., those interventions that address access and affordability to services) Future efforts need to examine the impact of the patient-provider relationship and the clinical setting on adherence, in addition to the generalizability of results to a wider range of populations	2005	9/11	Low- and middle-income countries (0) High-income countries (19): United States (12); Spain (2); France (2); Australia (2); Switzerland (1)
Crepaz et al. [45]^b^	Counselling Sexual health	To assess interventions for people living with HIV to determine their overall efficacy in reducing HIV risk behaviours and identify intervention characteristics associated with efficacy	The interventions were found to successfully reduce self-reported unprotected sex and STI acquisition, but not needle sharing, among PHAs Interventions with the following characteristics were found to significantly reduce unprotected sex: (1) guided by behavioural theory; (2) specifically focused on HIV transmission behaviours; (3) provided skills building; (4) delivered to individuals; (5) delivered by health-care providers or professional counselors; (6) delivered in settings where people living with HIV receive services; (7) delivered in an intensive manner; (8) delivered over a longer duration; (9) addressed a myriad of issues relating to coping with one’s serostatus, medication adherence, and HIV risk behaviours	2004	9/11	Low- and middle-income countries (0) High-income countries (14): United States (10); China (Hong Kong) (2); Canada (1); Netherlands (1); Not reported (1)
Crepaz et al. [38]^b^	Counselling Mental health Immune system functioning	To evaluate the efficacy of cognitive-behavioral interventions (CBIs) for improving the mental health and immune functioning of people living with HIV	PHAs who received CBIs showed a significant improvement in symptoms of depression, anxiety, anger, and stress, but not in immune functioning relative to controls No long-term evidence for significant intervention effects on symptoms of depression and anxiety, suggesting on-going behavioral reinforcement needed to prevent relapse CBIs for PHAs are more likely to achieve success if interventions incorporate stress management skills training and provide opportunities to practice skills	2005	8/11	Low- and middle-income countries (0) High-income countries (15): United States (11); China (Hong Kong) (2); Canada (1); Netherlands (1)
Simoni et al. [43]^b^	Counselling Adherence to highlight active retroviral therapy	To examine whether behavioral interventions addressing highly active antiretroviral adherence are successful in increasing the likelihood of a patient attaining 95% adherence or an undetectable HIV-1 RNA viral load	The most common intervention delivery method for HAART adherence was 1-on-1 counselling and group counselling, with the most common interveners being health care providers (physicians and nurses) or mental health counselors (psychologists) Participants who received an intervention were 1.5 times as likely to report 95 % adherence and 1.25 times as likely to achieve an undetectable viral load, relative to control participants Intervention effect sizes are: significantly stronger in studies that used a longer recall period (i.e., 2 weeks or 1 month) versus a shorter one (i.e., ≤7 days) for 95 % adherence; and larger in studies that provided didactic information on HAART and studies that included interactive discussions regarding adherence These findings suggest the importance of providing basic information to patients and engaging patients in discussions to help overcome cognitive factors (e.g., avoidance coping), lack of motivation, and unrealistic expectations about adherence behaviours	2005	8/11	Low- and middle-income countries (0) High-income countries (19): United States (14); France (2); Spain (2); Switzerland (1)
Moskowitz et al. [40]^b^	Counselling Social support Substance use	To determine which types of coping are related to psychological and physical well-being among people with HIV and whether contextual, measurement, or individual variables affect the extent to which coping is related to physical and psychological well-being	Direct action and positive reappraisal were consistently associated with better outcomes in PHAs across affective health behaviours, and physical health categories Disengagement forms of coping, such as behavioral disengagement and use of alcohol or drugs to cope, were consistently associated with poorer outcomes In some cases, coping effectiveness was dependent on contextual factors, including time since diagnosis and the advent of HAART	2005	5/11	Not reported
Johnson et al. [46]^a,b^	Counselling Health promotion Behavioural interventions HIV/AIDS information or education Sexual health/risk behaviour	To assess interventions to reduce HIV + individuals’ sexual risk	Behavioural interventions reduced sexual risk especially if they included motivational and skills components Such interventions have been less effective for older samples, suggesting the need for further refinement to enhance their efficacy Motivation and skill-based interventions have not yet been tested with HIV+ MSM who, in general, seem to have benefited less from extant risk reduction interventions	2004	5/10	Low- and middle-income countries (1): Tanzania (1) High-income countries (14): United States (14)
Weinhardt et al. [47]^b^	Counselling Sexual health/risk	To examine whether HIV counselling and testing (HIV-CT) leads to reductions in sexual risk behavior	HIV-CT appears to provide an effective means of secondary, but not primary, prevention of HIV infection Theory-driven research is needed to further explicate the determinants of behavior change in HIV-CT and empirically-driven research is needed to examine the effectiveness of specific counselling approaches with different content, modes of delivery, and levels of intensity HIV-CT is one part of an overall HIV prevention strategy that also includes individual-, community-, and policy-level interventions	1997	5/10	Low- and middle-income countries (6): Rwanda (2); Kenya (1); Zaire (1); Uganda (1); The Gambia (1) High-income countries (21): United States (18); Netherlands (1); Italy (1); Canada (1)
Simoni et al. [44]^a^	Counselling Health promotion Adherence to highly active retroviral therapy for pediatric infection	Adherence to antiretroviral therapy for pediatric HIV infection	Medication related factors significantly associated with adherence include: twice-per-day (vs 3-times per day) nelfinavir regimen; shorter length of time since treatment initiation; nelfinavir rather indinavir Patient related factors significantly associated with adherence include: Nonwhite (vs white) race; both younger and older age of child; children’s unawareness of their HIV diagnosis; beliefs regarding the positive impact of the medications on quality of life; lower intensity of alcohol use; housing stability; less depressive symptomatology; less child stress; decreased child responsibility for medications; improved health status/virologic or immunologic factors Caregiver/family related factors significantly associated with adherence include: Foster (vs biological) parent; higher self-efficacy; belief in the efficacy of the medication; less concern about hiding child’s diagnosis; better parent-child communication; less caregiver stress; higher quality of life; better caregiver cognitive functioning; better caregiver knowledge of antiretroviral medications; fewer barriers The review fails to provide definitive guidelines or to identify any gold standard for adherence assessment methods. The limitations of any single assessment strategy highlight the need to develop multi-systemic, cost-effective approach to assess and improve adherence to antiretroviral therapy for children with HIV	2005	4/10	Low- and middle-income countries (4): Brazil (1); South Africa (1); Cote d’Ivoire (1); Puerto Rico (1) High-income countries (28): United States (21); Italy (4); Belgium (1); Australia (1); Netherlands (1) Not reported (1)
Scott-Sheldon et al. [41]^b^	Counselling Mental health (depression, coping, stress management)	To examine the impact of stress-management interventions at improving psychological, immunological, hormonal, and other behavioral health outcomes among HIV positive adults	Stress-management interventions for HIV+ adults significantly improve mental health, fatigue and quality of life but do not improve stress, immunological or hormonal outcomes The absence of immunological or hormonal benefits may reflect the studies’ limited assessment period (measured typically within 1-week post intervention), participants’ advanced stage of HIV (HIV+ status known for an average of 5 years), the inclusion/exclusion of participants using ART, the lack of information regarding ART adherence, and/or sample characteristics Future research should examine more diverse samples and patient characteristics that might moderate intervention efficacy, in addition to using lengthier assessment periods to understand better the impact of stress-management interventions for HIV+ adults	2007	4/11	Not reported in detail (77 % of 35 articles were conducted in the United States)
Collins et al. [37]^a^	Counselling Health promotion Mental health Cognitive-behavioral therapy Psychotherapy	To examine the mental health risk factors for HIV, mental health consequences of HIV, psychosocial interventions of relevance for HIV-infected and affected populations	Stigma, disclosure and self-efficacy were found to have particular relevance for the successful implementation of these programs, in addition to economic factors Counselling and treatment teams should be aware of vulnerable periods in the course of HIV illness (e.g., periods of increased symptoms or pain) during which patients may have a greater need for support or be at greater risk for experiencing symptoms of mental illness. There is a need for methodologically sound studies of mental health throughout the course of HIV and interventions that employ identified variables (e.g., coping, family support) for efficacy in reducing symptoms of mental health	2005	2/10	Low- and middle-income countries (36): India (7); South Africa (5); Thailand (5); Brazil (4); Uganda (4); Kenya (2); Rwanda (2); Taiwan (2); Zaire (2); Zimbabwe (2); China (1); Costa Rica (1); Nepal (1); Russia (1); Tanzania (1); Trinidad and Tobago (1) High-income countries (3): Germany (2); China (Hong Kong) (1)

^a^We classified three reviews [37, 44, 46] as addressing both the counselling and case management and the health promotion domains but are only presented in this table

^b^These reviews conducted a meta-analysis as part of their analysis

**Table 2:** Included systematic reviews about health promotion

Review	Domain/topics studied	Focus of review	Key findings	Year of last search	AMSTAR (quality) rating	Countries in which included studies were conducted
O’Brien et al. [49]^a^	Health promotion Physical therapy Exercise	To examine the safety and effectiveness of aerobic exercise interventions on immunological/virological, cardiopulmonary and psychological parameters in adults living with HIV/AIDS	Performing aerobic exercise or a combination of aerobic exercise and resistive exercise for at least 20 minutes, at least three times per week for at least five weeks appears to be safe and may improve fitness, body composition, and well-being for HIV+ adults Statistically significant improvements were found for some outcomes of cardiopulmonary outcomes (VO2max), body composition (leg muscle area, percent body fat), and psychological status (depression-dejection subscale of the POMS) The review also found a trend towards potential clinically important improvements in cardiopulmonary fitness and psychological status; however, these findings should be interpreted cautiously due to missing follow-up data or the exclusion of exercisers who did not follow their regimen	2009	11/11	Not reported
O’Brien et al. [48]^a^	Health promotion Exercise	To examine the safety and effectiveness of progressive resistive exercise interventions on weight, body composition, strength, immunological/virological, cardiopulmonary and psychological parameters in adults living with HIV infection	Performing progressive resistive exercise or a combination of progressive resistive and aerobic exercise three times a week for at least four weeks appears to be safe and may lead to statistically significant and possible clinically important improvements in body weight and composition for medically stable adults living with HIV/AIDS	2003	10/11	Not reported
Mills et al. [50]	Health promotion Stress management Alternative and complementary therapy	To assess the effectiveness of complementary and alternative medicine treatments in HIV/AIDS and HIV-associated symptoms	Despite the widespread use of complementary therapies and alternative medicines by PHAs, few large-scale, methodologically sound clinical trials have been conducted to establish their effectiveness The majority of treatments tested in this review were supportive rather than curative in nature, with cognitive behavioural stress management therapies appearing to be the most promising treatment option for improving anxiety and quality of life	2004	6/11	Not reported
Crepaz et al. [52]^a^	Health promotion Sexual health Medication management	To determine whether (1) being treated with HAART, (2) having an undetectable viral load, or (3) holding specific beliefs about HAART and viral load are associated with increased likelihood of engaging in unprotected sex	HIV+ patients receiving HAART did not exhibit increased sexual risk behaviour whether their treatment achieved an undetectable viral load or not Beliefs about HAART and viral load were associated with unprotected sexual behaviour Disease severity beliefs and medical factors such as length of time receiving HAART and stage of disease may help explain increases in unprotected sexual behavior Recommended that HIV and STI patients should receive prevention messages emphasizing that having an undetectable viral load does not eliminate the possibility of transmitting HIV	2003	6/11	Low- and middle-income countries (0) High-income countries (24) United States (15); France (4); England (3); Australia (2); Canada (1); Netherlands (1); Switzerland (1) Not reported (1)
Malta et al. [53]	Health promotion Adherence to antiretroviral therapy	To identify factors associated with non-adherence to HIV treatment among HIV-positive drug users	Facilitators of HAART adherence among HIV+ drug users include access to drug abuse treatment (e.g., substitution therapy for opiate addiction), psychological characteristics (e.g., higher self-esteem, adherence self-efficacy), and access to mental health treatment Illicit stimulant use, social instability (e.g., unemployment, homelessness), and psychological problems (e.g., anxiety, depression) represents a key challenge for optimal adherence Review findings support the need for low-threshold/user-friendly health care delivery systems targeted to the specific needs of HIV+ drug users to optimize adherence, such as drug treatment, case-management, medical services and psychosocial supports	2007	4/11	Low- and middle-income countries (0) High-income countries (41) United States (22); Canada (8); France (6); Spain (3); Ireland (1); Italy (1)
Leaver et al. [51]	Health promotion Sexual health Adherence and access/utilization of health care	To assess the effects of housing status on health-related outcomes in people living with HIV/AIDS	Increased housing stability was significantly correlated with better health-related outcomes, as measured by medication adherence, utilization of health and social services, health status, and HIV risk behaviours The receipt of some form of housing assistance was found to be significantly associated with routine use of primary health care services, and housing instability was found to be a significant predictor of non-adherence to HAART	2005	4/10	Low- and middle-income countries (1) Cote d’Ivoire (1) High-income countries (28) United States (22); Canada (1); European Union (4) [France (1), Spain (1), Not reported (2)]; Australia (1)

Table 1 contains three reviews (each identified under footnote a) that address health promotion but are only presented in that table

^a^These reviews conducted a meta-analysis as part of their analysis

### Counselling and Case Management Reviews

The high quality reviews (those that received between 8 and 11 on the AMSTAR scale) focused on diverse topics. They included reviews of the setting and organization of care for PHAs [36], various mental health interventions for PHAs (including group psychotherapy and cognitive behavioral interventions) [37–41], interventions to address adherence to highly active anti-retroviral therapy (HAART) [42–44], and interventions to reduce PHA’s HIV risk behaviors [45–47]. The outcomes of these interventions varied depending on their focus. Some of the key findings from these high quality reviews highlighted their limitations. For example, Handford et al. [36] found that centralizing care in high concentration or high volume settings could lead to improved care outcomes for PHAs, but this evidence is mixed and limited to developed country settings. In addition, Handford et al. [36] found that case management was associated with improved outcomes but the limited number of studies and the varying definitions of case management leave considerable doubt about how best to implement such programs based on the studies reviewed. Another high-quality review by Himelhoch et al. [39], examined cognitive behavioral therapy, which was found to be efficacious in treating depressive symptoms among PHAs. However, the underrepresentation of women limited the generalizability of the findings [39]. Crepaz et al. [38] similarly found that PHAs who received cognitive behavioral interventions showed significant improvement in multiple mental health symptoms. However, immune functioning was not impacted, and the long-term intervention effects were uncertain. Interventions were more likely to achieve success if they incorporated stress management skills training and provided opportunities to practice skills [38].

High quality reviews about interventions to increase adherence to HAART indicated that these interventions were effective in increasing adherence. The characteristics that promote intervention success include delivery at the individual-level (as opposed to those delivered in groups), duration of 12 weeks or more, and interactive discussions about adherence [42, 43].

A high quality review by Crepaz et al. [45] about interventions to reduce PHAs risk behavior for HIV identified the following characteristics that significantly reduced unprotected sex: (1) guided by behavioural theory; (2) specifically focused on HIV transmission behaviours; (3) provided skills building; (4) delivered to individuals; (5) delivered by health-care providers or professional counselors; (6) delivered in settings where people living with HIV receive services; (7) delivered in an intensive manner; (8) delivered over a longer duration; and (9) addressed a myriad of issues relating to coping with one’s serostatus, medication adherence, and HIV risk behaviours [45].

The medium quality reviews (with scores between 4 and 7 on the AMSTAR scale) that addressed topics not covered by the high quality reviews focused on HIV testing and counselling [47] and stress management interventions [41]. Weinhardt et al. [47] found that HIV counselling and testing was effective for secondary prevention (i.e., early detection and treatment to limit disease progression and to prevent further HIV transmission) but not for primary prevention (i.e., preventing uninfected individuals from becoming infected). Scott-Sheldon et al. [41] found that stress management interventions impacted mental health symptoms, but not immunological functioning. This finding was similar to those in the high quality review by Crepaz et al. [38] which found that cognitive behavioral interventions improved mental health symptoms, but not immunological functioning.

A low-quality review [37] suggests that stigma, disclosure and self-efficacy are important factors to consider in psychosocial counselling interventions and that treatment teams should be aware of vulnerable periods in the course of HIV illness (e.g., periods of increased symptoms or pain).

### Health Promotion Reviews

The systematic reviews about health promotion (that did not also address counselling and case management) are included in Table 2. Two high-quality reviews found that sustained aerobic and progressive resistance exercise strategies may lead to clinically important improvements for people living with HIV/AIDS [48, 49]. Positive physical outcomes were observed in both reviews, and the aerobic exercise review also observed positive psychological outcomes.

A medium quality review by Mills et al. [50] assessed the effectiveness of complementary and alternative treatments, and found that mental health therapies (specifically, cognitive behavioural stress management therapies) appeared to be the most promising. A medium-quality review found a positive association between housing stability and better health-related outcomes [51]. The review also found that the receipt of some form of housing assistance was associated with routine use of primary health care services [51]. The review also found that housing instability was a significant predictor of non-adherence to HAART.

Across both domains, the most common areas of focus of the reviews were mental health interventions to support PHAs [37–41], and interventions to address adherence to HIV medications [42–44, 52, 53]. The highest quality reviews with a focus on mental health evidence suggest that cognitive behavioural interventions (including group therapy) were effective at improving symptoms of depression, anxiety and stress (but not immune functioning) [38, 39]. However, as outlined by Crepaz et al. [38], there is limited evidence about the long-term impact of these types of interventions. The highest quality reviews assessing adherence to HAART [42, 43] found that participants who received an intervention were 1.5 times as likely to report 95 % adherence and 1.25 times as likely to achieve an undetectable viral load. In addition, interventions targeting practical medication management skills, those targeting individuals versus groups and those delivered over 12 weeks or more were most effective at improving adherence. The most recent review, which was of medium-quality, found that drug abuse treatment, psychological characteristics (higher self-esteem) and access to mental health treatment facilitated better adherence to HAART [53].

## Discussion

Our overview was designed within the framework of helping Canadian national, provincial and local organizations meet their strategic goals related to program and policy development. The purpose of the scoping review was threefold: (1) to identify all systematic reviews related to counselling, case management and health promotion for PHAs, (2) to assess the quality and local applicability of the systematic reviews, and (3) to develop user-friendly summaries and disseminate them among program and policy decision-makers.

### Principal Findings

This overview found 18 systematic reviews (12 of which conducted a meta-analysis) addressing topics related to counselling, case management and/or health promotions for people living with HIV/AIDS. All of the systematic reviews except one were of medium- or high-quality and a user-friendly summary has been developed for each to support their use by health system stakeholders. The reviews addressed topics related to: setting and organization of care for PHAs; various mental health interventions for PHAs (including group psychotherapy and cognitive behavioral interventions); interventions to address adherence to highly active anti-retroviral therapy (HAART); interventions to reduce PHA’s HIV risk behaviors; aerobic and progressive resistance exercise; and housing stability.

Key findings from high-quality systematic reviews found research evidence to support: centralizing PHA care in high concentration or high volume settings; cognitive behavioural interventions for reducing symptoms of depression, stress and anxiety; interventions to promote adherence (particularly those that provide practical medication management skills, target individuals are delivered over a time-period of 12 weeks or more); and the use of aerobic and progressive resistance exercise.

### Strengths and Limitations of the Review

This overview of systematic reviews has several strengths. First, the methods used in the review are robust as they draw on validated search strategies for identifying systematic reviews and the objectives and process for selecting reviews followed an a priori protocol. Second, all of the included systematic reviews were quality appraised by two independent reviewers using a validated and commonly used tool. Lastly, in an effort to further support the use of the findings, we produced a user-friendly summary for each of the 18 included systematic reviews, which are available at (http://www.hivevidence.org/SHARE/ResourcesSummaries.aspx).

There are two main limitations to our review. First, our review is based on a search from 2009 and therefore may not include systematic reviews that have been completed since then (although we included updated versions of reviews that were originally caught in our search). Second, we conducted assessments of methodological quality of systematic reviews but not assessments of the strength of the evidence included within them. Readers should be aware that a systematic review of high methodological quality could have little utility in terms of the strength of the research evidence it includes. In other words, while a review may be well done, the studies available may be small and/or of low-quality. Lastly, though our process has made research evidence more accessible, decision-makers in community-based HIV/AIDS organizations do not have regular access to the online research databases where the full reviews are located. For example, though the user-friendly summaries provide crucial information in an accessible format, decision-makers may be unable to check the full reviews to clarify any specific issues.

### Implications of the Findings

This overview of systematic reviews provides a useful resource for supporting the development and delivery of evidence-informed support services in community settings. Service providers and policy makers can draw on the set of quality appraised and synthesized systematic reviews provided in this overview to rapidly determine whether there is any high-quality synthesized research evidence available about counselling, case management or health promotion for people living with HIV/AIDS. Researchers can use this set of systematic reviews to prioritize areas where updated systematic reviews are needed and work with service providers and policymakers to identify and prioritize areas for new systematic reviews. In addition, the findings from our synthesis also highlight the need to ensure consistent methodological standards in systematic reviews. Registering titles and protocols for systematic reviews and requiring specific quality standards as part of the registration process (as is done by the Cochrane Collaboration and PROSPERO) is a promising mechanism that may help increase the overall quality of reviews.

A remaining challenge or next step is to engage decision-makers in building their capacity to effectively use the available research evidence for program development purposes. Providing information, even in the form of user-friendly summaries, is helpful and necessary. However, a larger challenge is how to use the information in the context of reviewing, renewing or developing programs and policy. This speaks to the sustainability of locating, assessing, synthesizing and disseminating research evidence to decision-makers. Future efforts may examine the sustainability of mobilizing research evidence for decision-makers.

## Electronic supplementary material

Below is the link to the electronic supplementary material.

Supplementary material 1 (DOCX 15 kb)
