# Supplementary material for: Counselling, Case Management and Health Promotion for People Living with HIV/AIDS: An Overview of Systematic Reviews
Source: AIDS Behav. 2012 Sep 9;17(5):1612–25. doi: 10.1007/s10461-012-0283-1 (PMC3663251; doi:10.1007/s10461-012-0283-1)
Supplement: Supplementary file 1 — Supplementary material 1 (DOCX 15 kb) [file 10461_2012_283_MOESM1_ESM.docx]

**Appendix A – Search Strategy**

The following search strategy was used in Medline (OVID) in April 2009. The search was then adapted and applied to 11 other databases:

1. Embase
2. Cochrane Database of Systematic Reviews
3. International Bibliography of Social Science Abstracts
4. Social Science Abstracts
5. PsychInfo
6. Sociological Abstracts
7. Web of Science
8. Sociological Abstracts
9. Social Work Abstracts
10. CINAHL
11. Social Services Abstracts

In addition, while the search below was originally limited to English only articles, we later re-ran it to include publications in French.

1 exp hiv/ or exp hiv infections/ or HIV long term survivors/ or exp anti-hiv agents/ or exp hiv protease inhibitors/ or exp reverse transcriptase inhibitors/ or (hiv or human immunodeficiency or acquired immunodeficiency syndrome or acquired immune deficiency syndrome).tw. (247576)

2 aids.tw. (97809)

3 1 or 2 (268527)

4 exp counseling/ (25867)

5 exp psychotherapy/ (120070)

6 Case management/ (6369)

7 Patient care planning/ (28748)

8 exp patient care team/ (42502)

9 Health promotion/ (35050)

10 Community health planning/ (3629)

11 Community health services/ (22608)

12 Community mental health services/ (14461)

13 Social welfare/ (6574)

14 Counsel$.tw. (48914)

15 Pastoral care.tw. (487)

16 (Psychotherap$ or psychosocial therapy).tw. (25425)

17 Crisis intervention.tw. (1163)

18 Behav$ therapy.tw. (6718)

19 Cognitive therapy.tw. (1175)

20 Cognitive behav$ therapy.tw. (3903)

21 Cognitive behav$ technique$.tw. (156)

22 (Psychologic$ therap$ or psychologic$ treat$).tw. (2076)

23 Case manage$.tw. (6771)

24 care plan$.tw. (6083)

25 Health promot$.tw. (14527)

26 Wellness program$.tw. (413)

27 Health campaign$.tw. (548)

28 (Promot$ adj3 health).tw. (20310)

29 (Community health adj3 program$).tw. (612)

30 Community health service$.tw. (633)

31 Community mental health service$.tw. (494)

32 Support service$.tw. (2223)

33 (Aids services organization$ or aids services organisation$).tw. (2)

34 or/4-33 (346154)

35 3 and 34 (12402)

36 limit 35 to yr=1999-2009 (6345)

37 limit 36 to english language (5961)

38 limit 37 to "reviews (optimized)" (819)

39 from 38 keep 1-819 (819)

40 from 39 keep 1-10 (10)
